# Blood lactate predicts survival after percutaneous implantation of extracorporeal life support for refractory cardiac arrest or cardiogenic shock complicating acute coronary syndrome: insights from the CareGem registry

**DOI:** 10.1007/s11739-020-02459-0

**Published:** 2020-08-09

**Authors:** Italo Porto, Alessio Mattesini, Domenico D’Amario, Carlotta Sorini Dini, Roberta Della Bona, Marco Scicchitano, Rocco Vergallo, Antonio Martellini, Simona Caporusso, Carlo Trani, Francesco Burzotta, Piergiorgio Bruno, Carlo Di Mario, Filippo Crea, Serafina Valente, Massimo Massetti

**Affiliations:** 1 Dipartimento CardioToracoVascolare, IRCCS Ospedale Policlinico San Martino, Genova, Italy, Italian IRCCS Cardiovascular Network, Genoa, Italy; 2 Dipartimento di Medicina Interna e Specialità Mediche (DiMI), Università di Genova, Genoa, Italy; 3 Dipartimento del Cuore e dei Vasi, Azienda Ospedaliero-Universitaria Careggi, Florence, Italy; 4 Dipartimento di Scienze Cardiovascolari e Toraciche, Fondazione Policlinico A. Gemelli IRCCS, Rome, Italy, Italian IRCCS Cardiovascular Network and Università Cattolica del Sacro Cuore, Largo A. Gemelli, 00168 Rome, Italy; 5 Ospedale Sandro Pertini, Roma, Italy; 6 IRCCS Ospedale Pediatrico Bambino Gesù, Roma, Italy; 7 Spedali Riuniti, Leghorn, Italy; 8 U.O.C. Cardiologia Ospedaliera, A.O.U. Senese Ospedale Santa Maria Alle Scotte, Siena, Italy

**Keywords:** Extracorporeal life support, Cardiogenic shock, Cardiac arrest, Acute coronary syndrome

## Abstract

Refractory cardiogenic shock (RCS) or refractory cardiac arrest (RCA) complicating acute coronary syndrome (ACS) is associated with extremely high mortality rate. Veno-arterial extracorporeal life support (VA-ECLS) represents a valuable therapeutic option to stabilize patients’ condition before or at the time of emergency revascularization. We analyzed 29 consecutive patients with RCS or RCA complicating ACS, and implanted with VA-ECLS in two centers who have adopted a similar, structured approach to ECLS implantation. Data were collected from January 2010 to December 2015 and ECLS had to be percutaneously implanted either before (within 48 h) or at the time of attempted percutaneous coronary revascularization (PCI). We investigated in-hospital outcome and factors associated with survival. Twenty-one (72%) were implanted for RCA, whereas 8 (28%) were implanted on ECLS for RCS. All RCA were witnessed and no-flow time was shorter than 5 min in all cases but one. All patients underwent attempted emergency PCI, using radial access in ten cases (34.5%), whereas in three patients a subsequent CABG was performed. Overall, ten patients (34.5%) survived, nine of them with a good neurological outcome. Life threatening complications, including stroke (4 pts), leg ischemia (4 pts), intestinal ischemia (5 pts), and deep vein thrombosis 2 pts), occurred frequently, but were not associated with in-hospital death. Main cause of death was multi-organ failure. PCI variables did not predict survival. Survivors were younger, with shorter low-flow time, and with ECLS mainly implanted for RCS. At multivariate analysis, levels of lactate at ECLS implantation (OR 4.32, 95%CI 1.01–18.51, *p* = 0.049) emerged as the only variable that independently predicted survival. In patients with RCA or RCS complicating ACS who are percutaneously implanted with ECLS before or at the time of coronary revascularization, in hospital survival rate is higher than 30%. Level of lactate at ECLS implantation appears to be the most important factor to predict survival.

## Introduction

During last decades, the mortality rate of patients diagnosed with acute coronary syndrome (ACS) has been substantially reduced worldwide, due the combination of powerful pharmacological treatment with early revascularization, mainly employing percutaneous coronary intervention (PCI) [1, 2]. When ACS is complicated by refractory cardiogenic shock (RCS) or refractory cardiac arrest (RCA), however, the in-hospital mortality rate remains extremely high [3, 4]. In these critical conditions, mechanical circulatory support using veno-arterial extra-corporeal life support (VA-ECLS), able to provide haemodynamic stability during RCS or even total circulatory support during RCA, is considered appropriate in the most recent international guidelines, as ACS is classified as a potentially treatable etiology [5, 6]. Implantation of ECLS may be variously performed in the emergency department, in the intensive care unit, or in the catheterization laboratory, employing either a surgical or a percutaneous approach to cannulation, according to clinical presentation, available resources and local guidelines [7, 8]. Recently, for ACS patients, an emphasis shift has occurred, with several groups reporting relatively good outcomes for a team-based approach centered around the percutaneous suite, to provide rapid revascularization after the start of ECLS support [9, 10].

We report here the in-hospital outcome and factors associated with survival from two tertiary Italian centers who have adopted a similar, structured approach to ECLS implantation for ACS patients complicated by RCS or RCA, favoring percutaneous cannulation and aggressive revascularization by early PCI.

## Methods

### Patients

Data of patients undergoing ECLS due to RCS or RCA at two Italian tertiary care centers who have adopted a similar, structured, team-based approach to in-hospital ECLS implantation (the “Codice Viola” for the Gemelli Foundation Hospital, Rome, and the “ECMO Careggi Protocol” for the Azienda Ospedaliero-Universitaria Careggi, Florence) from January 2010 to December 2015.were prospectively entered into electronic hospital registries. Additional data required for this analysis were retrospectively evaluated. Patients who had undergone post-pericardiotomy ECLS implantation, who had ECLS implanted for non-cardiac causes, or for cardiac but non-ischemic etiology, or who had surgical cannulation, were excluded. We also chose not to include those patients who received an initial diagnosis of ACS, but in whom no coronary angiogram aimed at revascularisation was at least attempted, as we felt that those constituted a prohibitive risk subgroup.

### Definitions and protocols

Refractory cardiogenic shock (RCS) was defined as critical circulatory failure resulting in organ hypoperfusion unresponsive to conventional therapy with minimal chance of survival without ECLS. The likelihood of death in the absence of ECLS was deemed to be extremely high. RCA was defined by the lack of return to spontaneous circulation (ROSC) after 10 min of standard advanced cardiopulmonary resuscitation (CPR). No-flow time was defined by the duration of cardiac arrest before the start of effective CPR, low-flow time by the duration of cardiac arrest with low cardiac output during CPR and before ECLS implantation. In RCS, both protocols advised to consider only patients who had an increasing demand of inotrope and vasopressor doses and rising levels of serum lactate. For RCA, both hospital protocols advised to consider ECLS implantation only in witnessed CA with no flow time < 5 min, age ≤ 70 or ≤ 65 years, low flow time > 10 and < 60 min, and with end-tidal CO2 ≥ 10 mmHg, with one of the protocols also recommending as additional criteria < 3 adrenaline vials, arterial pH > 7.0 and arterial lactate < 20 mmol/L.

Contraindications to ECLS implantation were similar in both protocols and included: uncontrollable hemorrhage, irreversible neurological damage, severe trauma or known terminal malignancies, severe immunodepression or recent (within 30 days) organ transplantation, BMI > 40 or body weight > 140 kg, aortic dissection.

The decision regarding ECLS implantation was taken by the ECLS team that comprised an intensive care specialist, an interventional/intensive care cardiologist, a cardiac surgeon, and a perfusionist. Detailed sets of clinical and functional parameters including the SAVE score [11] were repeatedly assessed. Further, all patients underwent continuous control of routine laboratory parameters, including serum lactate. Left ventricular ejection fraction was assessed by transthoracic echocardiography.

### ECLS implantation

In the selected cases, ECLS was implanted percutaneously. Cannulation occurred in the emergency department, in the intensive care unit or in the catheterization laboratory. The Seldinger technique was employed and cannula sizes were selected according to the patient’s body weight, with arterial cannula 17–21 F and venous cannula 21–24 F. The equipment used heparinized polyvinyl chloride tubing, a membrane oxygenator (Quadrox Bioline; Jostra-Maquet, Orleans, France) and femoral cannulae (Biomedicus Carmed; Medtronic, Boulogne-Billancourt, France or) inserted percutaneously. A distal limb perfusion catheter (6–8 F) was consistently inserted in the superficial femoral artery to prevent lower limb ischaemia. Unfractionated heparin (70 U/kg body weight) was administered and the pump blood flow was initially set at 3–4 L/min. All patients were supported with mechanical ventilation.

### Outcome definition

The primary outcomes were in-hospital survival rate and good neurological outcome at discharge, which was assessed according to the cerebral performance category (CPC) scale 1–2 [12]. APACHE II score and SAVE-score were used for risk assessment [11, 13]. BCIS-JS [14] and Syntax [15]score were used to assess angiographic grading of complexity of coronary artery disease.

### Statistical analysis

Variables are expressed as a number and a percentage of patients. Continuous data are reported as median with corresponding interquartile deviation (IQR). The patients were divided into two groups: ‘survivors’ (survivors at the time of discharge) and ‘non-survivors’ (patients with in-hospital death). Univariate and multivariate logistic regression analyses were performed to identify predictors of in-hospital mortality. All variables with a *p* value < 0.1 in the univariate analysis were inserted into a forward stepwise multivariate model. Each statistical test was performed with SPSS (SPSS, IBM, Armonk, NY, USA software).

## Results

**Fig. 1:**
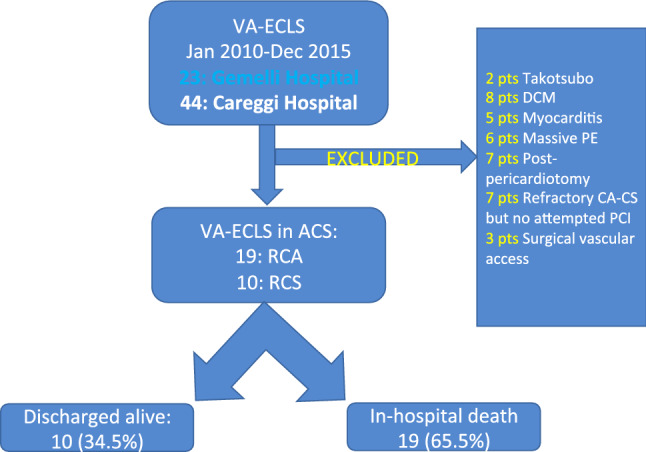
patients’ flow

From January 2010 to December 2015, 29 patients underwent ECLS implantation due to refractory cardiac arrest and cardiogenic shock complicating ACS. Details of the patients’ flow are represented in Fig. 1. Clinical characteristics are summarized in Table 1 and angiographic/procedural variables are reported in Table 2. All patients underwent attempted emergency PCI, using radial access in 10 (34.5%) cases, whereas in three patients CABG was subsequently performed. In 5 (17.2%) cases the PCI failed, as patency of the culprit vessels could not be obtained.

**Table 1:** Clinical characteristics

	Survivors 10 (34.5%)	Non-survivors 19 (65.5%)	*p* value
Age (years), mean (SD)	53.6 ± 5.7	60.9 ± 9.2	0.03
Male gender, *n* (%)	8 (80)	17 (90)	0.59
Body mass index (BMI), mean, kg/m^2^	27.7 ± 3.5	26.6 ± 1.9	0.4
Diabetes mellitus, *n* (%)	8 (80)	14 (73.7)	0.7
Hypertension, *n* (%)	5 (50)	9 (47.4)	0.9
Hyperlipidaemia, *n* (%)	4 (40)	4 (21)	0.3
Smoking,n (%)	6 (60)	8 (42.1)	0.3
COPD, *n* (%)	1 (10)	2 (10.5)	0.9
Chronic renal failure, *n* (%)	1 (10)	3 (15.8)	0.6
Previous CAD, *n* (%)	3 (30)	2 (10.5)	0.2
Previous PCI, *n* (%)	3 (30)	2 (10.5)	0.2
Chronic heart failure, *n* (%)	2 (20)	2 (10.5)	0.5
Peripheral artery disease, *n* (%)	1 (20)	4 (21)	0.4

**Table 2:** Angiographic/procedural characteristics

	Survivors 10 (34.5%)	Non-survivors 19 (65.5%)	*p* value
Coronary artery disease			0.74
1-vessel stenosis, *n* (%)	1 (10)	4 (21.0)	
2-vessel stenosis, *n* (%)	4 (40)	6 (31.6)	
3-vessel stenosis, *n* (%)	5 (50)	9 (47.4)	
Thromboaspiration, *n* (%)	4 (40)	10 (52.6)	0.5
PCI: Left main, *n* (%)	4 (40)	7 (36.8)	0.8
PCI: Left Anterior Descending, *n* (%)	9 (90)	11 (57.8)	0.07
PCI: Circumflex, *n* (%)	7 (70)	8 (42.1)	0.1
PCI: Right coronary, *n* (%)	1 (10)	8 (42.1)	0.07
Number of implanted Drug Eluting Stent, median (IQR)	2 (1–4)	1 (0.5–2)	0.08
Bifurcation PCI, *n* (%)	5 (50)	7 (36,8)	0.5
PCI Failure, *n* (%)	1 (10)	4 (21)	0.4
Complete revascularization, *n* (%)	1 (10)	6 (31.5)	0.2
CABG, *n* (%)	2 (20)	2 (10.5)	0.5
Autopulse, *n* (%)	0 (0)	4 (21)	0.1
Radial access, *n* (%)	5 (50)	5 (26.3)	0.2
Anti IIb/IIIa, *n* (%)	7 (70)	4 (21)	< 0.01
Antegrade limb perfusion, *n* (%)	9 (90)	18 (94)	0.6
BCIS-JS pre	11.6 ± 2.3	11.9 ± 5.3	0.6
Syntax score pre	30.5 ± 9.3	26.4 ± 12.4	0.4
BCIS-JS post	4 ± 3.1	3.8 ± 3.8	0.9
Syntax score post	8.9 ± 9.5	11 ± 14.3	0.7
Euroscore	17.1 ± 9.2	19.1 ± 9.7	0.6

All patients underwent ECLS implantation within 24 h from developing hemodynamic instability. Twenty-one (72%) were implanted for RCA, whereas 8 (28%) were implanted on ECLS for RCS. All RCA were witnessed, and no-flow time was shorter than 5 min in all cases but one. ECLS implantation was performed in the catheterization laboratory for 23 (79%) patients (of these, three in spoke hospitals), three patients underwent ECLS implantation in the intensive care unit, three in the emergency room. In 18 (62%) patients the hemodiafiltration (CVVHDF) was associated to the VA-ECLS, three of them already suffering from chronic renal failure. Among the complications, the most frequent were those related to bleeding at the access site of the VA-ECLS (Table 3). Twenty-one (72%) patients required transfusion of more than 1 unit of red blood cells, and in 13(45%) of them it was necessary to use more than eight red blood cells units. No cases of heparin-induced thrombocytopenia were observed. Severe infections, occurred in 15 (52%) patients, deep vein thrombosis in two, lower limb ischemia in four, intestinal ischemia was detected in five patients by autopsy, and cerebral ischemia occurred in four patients.

**Table 3:** Results summary

	Survivors 10 (34.5%)	Non-survivors 19 (65.5%)	*p* value
Indication for ECMO implantation			< 0.01
Refractory cardiac arrest, *n* (%)	4 (40)	17 (89.5)	
Refractory cardiogenic shock, *n* (%)	6 (60)	2 (10.5)	
Diagnosis			0.01
STEMI with RCA, *n* (%)	1 (10)	14 (73.7)	
ACS with RCA, *n* (%)	2 (20)	2 (10.5)	
STEMI with RCS, *n* (%)	4 (40)	2 (10.5)	
ACS with RCS, *n* (%)	3 (30)	1 (5.3)	
Low-flow time (min), median (IQR)	45 (28–65) (*n* = 4)	60 (50–70) (*n* = 17)	0.28
Low flow time < 30 min, *n* (%)	2/4 (50)	16/17 (94.1)	0.02
SAVE score, mean	− 5.3 (4.1)	− 8.2(3.7)	0.08
IABP, *n* (%)	10 (100)	15 (78.9)	0.1
CVVH-DF, *n* (%)	5 (50)	13 (68.4)	0.2
Hypotermia, *n* (%)	4 (40)	13 (68.4)	0.1
Inotropic agent			
Norephinephrine, *n* (%)	9 (90)	19 (100)	0.2
Ephinephrine, *n* (%)	2 (20)	6 (31.5)	0.5
Dopamine, *n* (%)	8 (80)	13 (64)	0.7
Dobutamine, *n* (%)	2 (20)	6(31.5)	0.5
Levosimendan, *n* (%)	8 (42.1)	6 (60)	0.2
VIS score			
0 h, median (IQR)	36.2 (39)	42.5 (44.5)	0.9
24 h, median (IQR)	25 (20.7)	40 (33)	0.1
48 h, median (IQR)	21 (22.4)	24 (33)	0.5
ECLS weaning, *n* (%)	10 (100)	1 (5.2)	< 0.01
ECLS duration (days), median (IQR)	3.8 (3.5)	5 (3)	0.5
White blood cells (/mm^3^)	27,375 ± 4612	16,120 ± 7545	0.6
Hb admission (g/dl)	11.5	10.1	0.25
Hb nadir (g/dl)	7.7	6.9	0.19
eGFR admission (ml/min)	77.9	54.2	0.03
pH admission	7.3	7.2	0.3
pH 24 h	7.3	7.6	0.01
Lactate at admission (mmol/l)	4.3	11.2	< 0.01
Lactate at ECLS (mmol/l)	5.4 (1.8–6.4)	10.2 (7.8–15)	< 0.01
Lactate 24 h after ECLS (mmol/l)	2.5	5.7	< 0.01
Complications			
Stroke, *n* (%)	2 (20)	2 (10,5)	0.5
Leg ischaemia, *n* (%)	0 (0)	4 (21)	0.1
Intestinal ischaemia, *n* (%)	2 (20)	3 (15.7)	0.7
Deep venous thrombosis, *n* (%)	2 (20)	0 (0)	
Blood transfusion, *n* (%)	9 (90)	16 (84.2)	0.8
Blood transfusion units	16	11	0.2

Weaning from VA-ECLS was obtained in 11 (38%) patients. Only one patient weaned from VA-ECLS did not survive the explant and died due to cerebral ischemia after weaning. Finally, 10 (34%) patients were discharged alive.

Overall, survivors were younger, with shorter low-flow time, and with ECLS mainly implanted for RCS, as opposed to RCA. Main cause of death was multi-organ failure (MOF), occurring in 9/19 (48%) ‘non-survivor’ patients; cardiogenic shock was the cause of death in 5 (26%) cases. The remaining 5 (26%) patients died due different causes: intractable ventricular fibrillation, ECLS access complications, pneumonia and hemoperitoneum, hemorrhagic shock, brain death and cerebral ischemia.

At univariate regression analysis, age and blood lactates at the time of ECLS implantation, emerged as predictors of mortality. PCI variables and the VIS score (evaluating the strength of administered inotropics agents) did not predict survival. At multivariate analysis levels of lactate at ECLS implantation (OR 4.32, 95% CI 1.01–18.51, *p* = 0.049) emerged as the only significant variable to predict survival.

## Discussion

The main findings of the present study are:

1. In-hospital survival rate of more than 30% is observed for patients with CS or CA complicating ACS in tertiary Italian hospitals that have adopted a similar, structured approach to ECLS implantation, and in patients in whom PCI was at least attempted.
2. Serum lactate measured at the time of VA-ECLS implantation, an index of profound haemodynamic derangement, is an independent risk factor of mortality in those patients, whereas procedural and angiographic variables, as well as complications occurring during hospital stay, do not seem to predict survival in our cohort.

Regarding the first point, the 34% survival to discharge observed in our cohort is in line with data from the ELSO cohort, the largest database available, which showed a survival to discharge of 38% in adult patients receiving ECLS for CS or refractory CA of different etiologies [16].

In the more selected case of CA or CS complicating ACS, the reported survival to hospital discharge varies from 33% in a Japanese cohort of 98 patients [17], to 39% of a French cohort of 77 patients with CS [18], to 67% of an American cohort of 18 patients with CS [10], with a recent systematic review and metanalysis including 739 patients from 17 studies reporting a short-term survival (defined as absence of death occurring during hospitalization or in the first month) of 42%. Whereas several confounding factors (including small sample sizes and differences in inclusion criteria) may explain this variability in survival to discharge, our study can be more closely compared with two recent reports, not included in the previous metanalysis.

A 29-patient cohort from Switzerland has been recently described, reporting a mortality of 62.1% at 30 days. In these patients, blood lactate peak in the first 24 h was markedly increased in non-survivors, and the peak of blood lactate > 11 mmol/l independently predicted 30-day mortality [19]. Differently from our cohort, however, a full percutaneous technique for ECLS implantation was only utilized in 10 (55.6%) patients of the non-survivors group and in 9 (81.8%; *p* = 0.093) of the survivors.

Another, larger (83 patients) cohort was reported by De Waha, comprising patients undergoing fully percutaneous ECLS implantation performed by interventional cardiologists for refractory CS. Etiology was MI in 63.9%, acute deterioration of ischaemic cardiomyopathy in 6.0%, non-ischaemic acute heart failure in 16.9%, valvular heart disease in 9.6%, and interventional complications in 3.6%. Of note, and despite the exclusion of refractory CA and the variable etiology, in-hospital mortality was remarkably similar to our sample (68.7%) [9].

It would appear, thus, that the short-term prognosis of patients with refractory CS or refractory CA remains relatively poor (but not dismal) in different countries and variable settings, highlighting the need for better patient selection and uniformization of ECLS inclusion and management protocols.

Regarding the value of biomarkers to gauge the prognosis of such patients, it should be noted that the main mechanism of hyperlactatemia is related to the development of anaerobic metabolism [3, 20, 21]. The assessment of lactates in patients with shock has been performed for > 30 years [22]. Several studies investigating the association of serum lactate and in-hospital survival in CA treated conventionally (i.e. without circulatory assist device) established a predictive link between survival and initial lactate levels and reported serum lactate as an independent prognostic factor of mortality and neurological outcome [23, 24]. Moreover, recent retrospective studies [19, 24–27] (the largest including 117 refractory CA [26]) revealed serum lactate as a potent predictor of outcome for patients treated with ECLS. Additionally, even if changes in lactate take place more slowly than changes in systemic arterial pressure or cardiac output, lactate levels decrease over a period of hours with effective therapy [20]. In patients suffering from CS and CA, the rate of clearance of excess serum lactate is also associated with VA-ECLS efficacy on organ perfusion [28]. Lactate cut-off values (pre-ECLS blood lactate greater 10 mmol/l, higher than 12 mmol/l 3 h after ECLS implantation, higher than 7.05 mmol/l at 6 h and 4.95 mmol/l at 12 h) have been suggested to predict the occurrence of multiorgan failure or inadequate tissue perfusion in this context [17, 19, 20].

## Conclusion

Additional criteria to guide ECLS initiation are urgently needed for better patient management: implementation of a simple, easily measured index such as serum lactate may potentially add to our armamentarium of tools for predicting futility in this extreme context.
